# Toxic External Exposure Leading to Ocular Surface Injury

**DOI:** 10.3390/vision7020032

**Published:** 2023-04-03

**Authors:** Steven H. Rauchman, Brandon Locke, Jacqueline Albert, Joshua De Leon, Morgan R. Peltier, Allison B. Reiss

**Affiliations:** 1 The Fresno Institute of Neuroscience, Fresno, CA 93730, USA; 2 Department of Medicine and Biomedical Research Institute, NYU Long Island School of Medicine, Mineola, NY 11501, USA; 3 Department of Psychiatry and Behavioral Health, Jersey Shore University Medical Center, Neptune, NJ 07753, USA

**Keywords:** eyes, toxicity, vision, cornea, pesticides, ocular inflammation, pollution, chemical injury, particulate matter

## Abstract

The surface of the eye is directly exposed to the external environment, protected only by a thin tear film, and may therefore be damaged by contact with ambient particulate matter, liquids, aerosols, or vapors. In the workplace or home, the eye is subject to accidental or incidental exposure to cleaning products and pesticides. Organic matter may enter the eye and cause infection. Ocular surface damage can trigger a range of symptoms such as itch, discharge, hyperemia, photophobia, blurred vision, and foreign body sensation. Toxin exposure can be assessed clinically in multiple ways, including via measurement of tear production, slit-lamp examination, corneal staining, and conjunctival staining. At the cellular level, environmental toxins can cause oxidative damage, apoptosis of corneal and conjunctival cells, cell senescence, and impaired motility. Outcomes range from transient and reversible with complete healing to severe and sight-compromising structural changes. Classically, evaluation of tolerance and safety was carried out using live animal testing; however, new in vitro and computer-based, in silico modes are superseding the gold standard Draize test. This review examines how environmental features such as pollutants, temperature, and seasonality affect the ocular surface. Chemical burns to the eye are considered, and approaches to protect the ocular surface are detailed.

## 1. Introduction

A multitude of chemicals are patented each year and millions of others are commercially available, but the extent of their toxic effects on the human eye are unclear [1,2]. Chemical exposure can occur through a variety of routes, including inhalation, transdermal, and ingestion, but exposures through the eyes are particularly dangerous. Even short-term exposures to small amounts of some chemicals can result in eye injury, vision loss, and permanent disability. In a sample of 900 emergency rooms across the U.S., there were 144,149 eye injuries over a 3-year period and $106 million in emergency department costs alone [3]. Although many injuries were work-related, most were in children or occurred in residential settings where safety concerns are not routinely addressed. Given the vulnerability of the eyes to damage by chemicals, the ocular surface has been widely used historically to test the potential for chemical substances to cause injury [4]. In this review, we discuss classical tests using animal models and their successors such as in vitro cultures as well as new, in silico methods that employ computer modelling to estimate the extent to which novel chemicals damage the eye. We then discuss how air pollutants, pesticides, cleaning products, and other materials may damage ocular surfaces. We cover the treatment and preventive measures that may minimize or avoid long-term visual compromise.

## 2. Assessments of Ocular Toxicity

### 2.1. The Draize Eye Test

The Draize eye irritation test was developed by the Food and Drug Administration (FDA) to assess the potential ocular toxicity of products, including cosmetics, insecticides, hair products, and sunscreens that were likely to come in contact with the eye during routine usage by the typical consumer [5]. The test entails the exposure of one eye from each of three to six rabbits to a dosage of 0.1 mL or 0.1 g of the liquid or solid substance being studied [6]. The focus of instillation is the lower conjunctival cul-de-sac of the rabbit eye [7]. Effects on the conjunctiva, cornea, and iris, ranging from slight, reversible irritation to severe, irreversible irritation, and vision loss are observed and recorded based on a subjective scoring system [8]. However, the “score” assigned to a chemical would be mainly associated with the degree of corneal injury and opacity present (80 points), with conjunctival irritation (20 points) and inflammation of the iris (10 points) being measured with lesser value on the overall “Maximum Average Score” determined from the average of the scores from each rabbit [7]. Observations of eye irritation take place at specific intervals: 1, 24, 48, and 72 h, and 7 days after applications [9]. 

Evaluating ocular toxicity by exposing the eye of an experimental rabbit was thought to be a reasonable model for the human eye. Also, while the reliance of the Draize test on subjective scoring of toxicity introduced some variability, it could prevent serious toxic exposure of a product before it reached the marketplace. These animal-based models raised much public concern given the potential for the animals to feel pain for days on end during testing of a hazardous substance. Routine cosmetic testing has become increasingly undesirable as public awareness of animal welfare issues has grown, leading manufacturers to seek out types of testing that are more humane and less expensive [10,11]. 

### 2.2. In Vitro Testing: Reconstructed Human Cornea-like Epithelium (RhCE)

Although incapable of replacing the Draize test entirely, in vitro tests have largely supplanted the Draize test as they are simple, reproducible, and inexpensive indicators of ocular toxicity [12]. The usage of human cell cultures from the corneal epithelium in many in vitro models allows for an accurate representation of the in vivo human response to toxic substances. These human corneal cells construct a three-dimensional epithelial model [13]. Time-to-toxicity measurements (ET50) provide the time required for the cell or tissue viability to experience a 50% decrease after exposure, and can be used to classify the cytotoxicity of the substance of interest [14]. The limited availability of human corneal epithelial cells for culture has led to the development of rabbit corneal epithelium for in vitro models [15]. 

The 2 validated RhCE models are EpiOcular™ and SkinEthic™ and they are quite similar with the exception of the type of cell used. EpiOcular™ utilizes primary epidermal keratinocytes derived from human foreskin and cultured in serum-free media to resemble corneal epithelium while SkinEthic™ uses immortalized human corneal epithelial cells [16,17]. 

The EpiOcular™ Eye Irritation Test (EIT), an in vitro 3D epithelial model, is commercially available from the MatTek Corporation. The EIT relies upon normal (non-transformed) human cells grown to form a stratified, squamous epithelium [18,19]. After a substance is applied to the model, the percent viability of the cell culture is commonly determined using an assay, often the 3-(4,5-dimethylthiazol-2-yl)-2.5-diphenyltetrazolium bromide (MTT) assay to test for cytotoxicity, where the MTT is reduced to formazan crystals by the mitochondria of the living cells. A highly cytotoxic irritant results in a loss of viability of the culture to 60.0% or less, whereas a viability in excess of 60% relative to a negative control suggests that the chemical is a non-irritant [16,20]. Others have used another viability assay, the lactate dehydrogenase (LDH) leakage assay to evaluate toxicity of chemicals. It is based on the release of the cytosolic LDH enzyme into extracellular medium by dead cells where its activity can be measured [21]. ET50 values can be measured with MTT or LDH viability assays to determine relative cytotoxicity via comparisons with in vivo animal data [22,23]. These MTT and LDH cytotoxicity tests are indicators of reductions in cell viability. A greater speed and depth of injury or decline in cell viability from a substance denotes greater cytotoxicity [15,24]. Cytotoxicity corresponds to the ocular irritancy of the substance. 

The EIT is often applied to products in the cosmetic, household, personal care, and industrial chemical industries [25]. The EpiOcular™ EIT is not intended to differentiate between Globally Harmonized System of Classification and Labelling of Chemicals (GHS) Category 1 (severe, irreversible irritation and serious eye damage) or GHS Category 2 (reversible eye irritation). It can, however, distinguish non-irritants (no category, not requiring classification) from irritants requiring classification [26]. 

Another alternative to the Draize test, the 3D HCE model developed by SkinEthic™ Laboratories. This system consists of immortalized human corneal epithelial cells in a chemically defined medium that structurally resembles the corneal mucosa of the human eye [27,28]. Percent viability is quantified after a single chemical exposure based on the MTT assay and compared with an unexposed control [29]. Like the EpiOcular™ system discussed above, the HCE model is also incapable of assigning substances to Category 1 or Category 2 of the GHS [29,30]. Despite this constraint, a viability above 60% after exposure to a liquid or a viability above 50% after exposure to a solid is designated “No Category”, or non-irritation [29,31]. The SkinEthic™ HCE model is utilized to evaluate the raw materials and products of cosmetic, chemical, and pharmaceutical companies [25]. 

Despite their limitations for use in classifying chemicals according to the GHS categories, recent publications have suggested that when applying this model in a time-to-toxicity approach, these systems are valid for predicting GHS categories [30,32].

### 2.3. In Silico Models 

Over the past decade, there has been great interest in using advances in computer science to predict the potential for chemical substances to do harm. These in silico models use known relationships to predict and simulate the potential ocular toxicity of previously untested substances [33,34]. In particular, quantitative structure–activity relationships (QSAR) predict ocular toxicity from the relationship between chemical structure and biological effect or activity of the sample, as the activity of a molecule is a reflection of its structure [35]. The QSAR model utilizes molecular descriptors derived from atomic or molecular properties to then mathematically relate variations in a substance’s molecular framework or general properties to levels of activity and toxicity [36,37]. These models of ocular toxicity are thus created based on relationships of preexisting data, eliminating the requirement of experimentation. The limits of computer modeling should always be understood when it relates to human safety [38]. 

Ultimately computers can only manipulate data, but they do not create it. Although QSAR models provide rapid, computer-generated relationships, they rely on high quality databases to produce accurate assessments of ocular toxicity [34,39]. Nonetheless, such algorithms and equations in the QSAR model can display these structure–activity relationships without direct testing on animal cells avoiding standardization, replication and welfare issues that accompany the use of bioassays and animal models; while, greatly reducing the time and cost of testing new compounds.

## 3. Pollution Effects

It is widely understood that air pollutants have deleterious effects on human health and have been linked to increased morbidity and reduced life expectancy [40]. Prevalent air pollutants that have been linked to such disease outcomes include ozone, particulate matter, carbon monoxide and carbon dioxide (CO and CO_2_), and nitrogen oxides (NOx) [41,42,43]. Airborne particulate matter can be subdivided into fine and coarse fractions. Fine particulate matter is characterized by aerodynamic diameter of 2.5 microns or less (PM2.5), while coarse particulate matter has aerodynamic diameters less than 10 microns and greater than 2.5 microns (PM10). 

The adverse health outcomes of air pollution are worsening as pollutants continue to be released into the atmosphere from motor vehicles and other sources [44,45,46]. As with other chemicals, the eyes are highly vulnerable to airborne pollutants due to the thin nature of the precorneal tear film that shields the cornea from environmental hazards [47]. Though it is difficult to separate the consequences of each individual air pollutant on different aspects of eye health, isolated scientific studies have correlated each pollutant with pathogenic processes. We will discuss component pollutants and their effect on the eye surface separately and then in aggregate.

### 3.1. Ozone

Ozone (O_3_) is a common gas pollutant in the atmosphere with oxidizing properties that incites inflammation and causes ocular surface disease [48,49]. It has been linked to several ocular surface disorders, including conjunctival chemosis, or inflammation of the eye membrane; conjunctival injection, or swelling of conjunctival vessels; and increased production of pro-inflammatory cytokines [50].

The toxicity of ozone can be attributed to its status as a very active free radical that facilitates the formation of reactive oxygen species (ROS) on the ocular surface, resulting in surface inflammation [51]. The accumulation of excess of ROS may overwhelm antioxidant defenses such as glutathione, leading to oxidative damage to the ocular surface, and tissue inflammation. Such accrual of oxidative damage has been implicated in several eye diseases, most notably in Dry Eye Disease [52].

### 3.2. Airborne Particulate Matter

Airborne particulate matter generally results from dust (coarse particulate matter) or vehicular and fuel exhaust (fine particulate matter) [53]. Thus, coarse and fine particulate matter are made of different primary components.

Fine particulate matter has a very complex and heterogeneous chemical composition, consisting of particles of carbon-containing organic matter, elemental carbon, sulfate, nitrate and ammonium salts, polycyclic aromatic hydrocarbons, metal elements, and mineral dust [54,55]. Coarse particulate matter is composed of dust, calcium, carbon, silica and organic matter [56,57]. Both PM10 and PM2.5 are of key epidemiological and mucosal interest due to their small size and resultant ability to penetrate epithelial and mucosal surfaces and both laboratory and epidemiological studies support that PM may have a role in ocular surface disease [58,59]. 

Exposure of mice to fine particulate matter (PM2.5) resulted in dry eye syndrome, as evidenced by increased inflammation in the cornea and conjunctiva, increased tear film damage, the induction of apoptosis in corneal superficial and basal epithelium, and decrease in tear volume [60]. These results were consistent with results of other studies that found reductions in tear volume, increases in corneal irregularities, and decreases in stability of tear film due to deficits in the mucin-4 layer of the film in mice that were exposed to particulate matter [61,62]. Yang et al. placed eye drops with increasing concentration of fine PM into the right eye of C57BL/6 mice and found a dose-dependent decrease in tear secretion and conjunctival goblet cells, consistent with findings in dry eye in humans [63]. In both conjunctival and corneal tissues of the PM-treated eye, cytokines IL-18, IL-22, IL-23, and MCP-1 were increased after 6 months of exposure. Increased apoptosis was also detected on the conjunctival surface in these mice. Additional studies with cultured human corneal epithelial cells also suggest that PM may result in eye damage. Yang et al. [63] found increased ROS production after exposure to 0.1 mg/mL and 0.2 mg/mL of fine PM over 12 h and 24 h. A transcriptomics analysis found that the mRNA expression profile of PM2.5 exposed cells differed significantly from that of unexposed control cells, notably in the expression of 65-long non-coding (lnc)RNAs [64]. Functional mapping of the lncRNAs differentially produced with fine particulate matter exposure suggested that PM2.5 may activate pathways linked to cancer, RNA transport, and the small GTPase Ras-associated protein-1, which is involved in cellular signaling. Taken together, the results of these studies suggest a clear toxicity of fine particulate matter to the ocular surface, causing cytokine production as well as cellular damage and death.

Diesel exhaust is worth special mention because, in addition to a mixture of gasses, it contains fine particulate matter less than 1 µm in diameter that is considered to have high toxicity and carcinogenicity [65,66,67]. Diesel exhaust nanoparticles cause ocular surface disruption and corneal and conjunctival inflammation in a murine model and an inflammatory response in cultured human conjunctival epithelium [68,69]. These very small particles also decrease viability and proliferation of human corneal and conjunctival epithelial cell lines [70]. Limiting diesel emissions via government regulation can reduce particulates in the environment.

Epidemiological studies suggest that PM10 exposure may also increase the risk of several ocular surface diseases, including childhood glaucoma, conjunctivitis, and keratitis [71,72,73]. An 11-year study conducted on a cohort of infants found that exposure to airborne particulate matter, particularly PM10, was correlated with increased diagnoses of childhood glaucoma [74]. A study conducted on a population of 769 individuals in Korea found that the incidence rate of conjunctivitis and keratitis was elevated for those residing in regions in the 80th percentile for PM10 concentrations as compared to regions in the 20th percentile, with number of conjunctivitis and keratitis patients 0.10 per 1,000 ER patients and 0.05 per 1,000 ER patients, respectively [75].

### 3.3. Nitrogen Oxides

Although there are physiological roles for nitric oxide (NO) and all 3 NO synthetase isoforms are expressed in the eye, nitrogen oxides may also have damaging effects on the ocular surface [76,77]. Of the members of the NOS family (endothelial NOS (eNOS), neuronal NOS (nNOS) and inducible NOS (iNOS)), iNOS is the isoform associated with inflammation.

Exposure to abnormally high levels of NO gases have been linked to the progression of ocular surface disorders such as dry eye, conjunctivitis, pterygium, corneal neovascularization, and microbial keratitis [78,79]. In a bimodal pattern, low levels of NO may promote corneal healing while high levels are destructive [80]. It has been postulated that cellular damage resulting from high levels of NO exposure is a result of the interactions of the highly reactive gas with ROS such as superoxides [81,82] In this model, the NO and superoxide form oxygen species that have even higher toxicity, including peroxynitrite, which causes damage through lipid peroxidation and tyrosine nitration of proteins [83,84].

A correlation between NO_2_ exposure and worsening dry eye syndrome was found in a large population-based study from Korea [85]. These findings were corroborated in a study conducted in the city of São Paulo, Brazil in which Novaes and colleagues investigated the effects of traffic-related air pollution on the ocular surface. They reported a correlation between NO_2_ and elevated scores in irritative dry eye symptoms, such as decreased tear break up times and increased instances of meibomitis [86,87].

### 3.4. Combined Pollutants

In day-to-day life, air pollutants are not found in an isolated manner; thus, the deleterious eye health consequences of each category of air pollutants must also be evaluated collectively. Population-based studies are particularly effective at assessing the risks of combinations of environmental pollutants on ocular surface disorders. They allow correlations to be made based on the types of pollutants to which subcategories of the population are more likely exposed and specific health outcomes. A study conducted by Malerbi and colleagues in São Paulo Brazil examined 200 patients with eyelid disease and found a significant correlation between levels of combustion-derived pollutants and clinical manifestations of blepharitis. Higher levels of vehicular emissions (PM10, NO_2_, and CO) were associated with increased eyelid debris and elevated meibomian gland secretion, considered markers of blepharitis in patients [88,89]. A population-based study from Taiwan found that outdoor air pollutants are linked to increased rates of age-related macular degeneration, especially for elevated quartiles of NO_2_ or CO [90]. A Delhi-based controlled study investigated the effect of environmental toxins on the ocular surface more broadly, noting that those exposed to higher degrees of pollutants related to vehicular exhaust had higher scores in ocular irritation and discomfort, as measured by redness and irritation surveys and Schirmer’s test results [91]. A cross-sectional study analyzing ophthalmologic outpatients in urban areas of China found a strong correlation between air pollutant exposure and increased incidence of dry eye disease, further supporting the notion that air pollutants irritate the ocular surface and cause increased risk of ocular surface disorder development [92]. Further studies are needed to determine whether or not combinations of different chemicals result in additive or synergistic impacts on eye health.

## 4. Air Bag Deployment

Air bags are a passive safety restraint designed to explosively inflate during automobile accidents and protect the driver and front-seat passenger from intracranial, upper extremity, and chest injuries by preventing violent contact with the steering wheel assembly, dashboard, or windshield of the car [93,94,95]. Although air bags are effective in preventing brain injuries, their rapid deployment places the eye at direct risk of injury from blunt trauma, despite lowering the likelihood and severity of orbital fracture [96]. Blunt ocular trauma can result from the impact between the vehicle occupant’s eye and the surface of the air bag while it inflates or after full expansion [97]. Blunt trauma can be associated with anterior segment injuries including corneal abrasion, hyphema, and chemical keratitis [98]. Corneal abrasions are a product of collisions between the corneal surface and the air bag fabric, which involve direct, rapid contact that can imprint the open eye onto the air bag during an automobile accident [97,99]. Although often reversible, the abrasions decrease the corneal endothelial cell count and corneal transplants may be required when damage is irreversible in various cases such as that of bullous keratopathy [100,101]. 

Hyphema is the visible accumulation of blood in the region between the cornea and iris, the anterior chamber, that is the result of the flattening of the anterior chamber from an increase in pressure upon impact [97,102,103]. This applied pressure from an object induces tearing and eventual leakage in blood vessels of the ciliary body and iris [104].

Chemical keratitis can also occur in patients who have experienced blunt ocular trauma in the form of burns. These burns are often caused by the emission of various chemicals that are required for expansion of the air bag; but, are toxic to the eye. Examples of such chemicals include sodium hydroxide, carbon dioxide, sodium bicarbonate, and metallic oxides, which compose the alkaline aerosol produced by the combustion of the solid propellant sodium azide [105]. This combustion reaction is responsible for the inflation of the air bag and release of high-temperature nitrogen gas and other byproducts [105,106]. Chemical keratitis particularly involves contact between the cornea and the alkaline aerosol, and subsequent injury and inflammation of the cornea corresponding to the duration of exposure [97,106]. Immediate irrigation of the injured eye is vital to reduce damage from the alkaline burn [107].

Additionally, blunt trauma from air bag inflation can be linked to posterior segment injuries, namely retinal tearing, and detachment [97,108]. These injuries are related to traction and distension in the vitreous base region during blunt trauma, which can apply pressure and thus cause breakage on the retina [109,110]. 

Ongoing efforts by engineers and healthcare professionals are needed to improve vehicle safety technologies to maximize protection and minimize injury for automobile occupants.

## 5. Pesticide Exposure 

### 5.1. Pesticide Overview

Pesticides are potent environmental pollutants that are especially relevant to workers in the agricultural industry, exterminators, and pesticide manufacturers [111]. Approximately 866 million workers are employed in agriculture worldwide representing about 20% of the world’s wage-earning labor force, making occupational exposure to pesticides a pressing global health concern [112,113]. Pesticide use has increased steadily, and exposure is a health concern for the general population since phenomena such as pesticide drift or the presence of residues in food or drinking water can have deleterious health consequences [114,115]. The reporting of pesticide exposure-related health concerns is complicated by the varying levels of toxicity of different agro-chemicals, as well as the variability in exposure level and route of exposure (ingestion, inhalation, skin, or mucous membrane absorption) [116]. 

Pesticides, categorized as insecticides, herbicides, and fungicides, are often composed of organophosphates, organochlorines, and carbamate compounds [117,118,119,120]. These classes of compounds interact with several cellular receptors and interfere with normal bodily function.

The health concerns related to pesticide exposure have been extensively documented, and chronic exposure to toxic pesticides has been linked to increased risk of cancer, dermatoses, and genotoxic, neurotoxic, and respiratory consequences [121,122,123]. Pesticide application leads to high levels of ocular exposure to toxic chemicals [124]. Pesticides can easily make their way into the eye from accidental splashing or by rubbing the eye with contaminated hands or cloths or by absorption from the air [125,126]. While exposure to pesticides is common, the impact of the ocular route of exposure and its consequences is poorly understood. Unfortunately, there is a gap in the medical literature regarding the effects of pesticides, especially pesticides of different classes, on the ocular surface. 

### 5.2. Herbicides and Insecticides

The herbicide paraquat, an organochlorine dipyridylium quaternary ammonium salt, is used frequently in agricultural fields and is known to be toxic to the ocular surface. Paraquat has been banned in European Union since 2007. Its toxicity is believed to relate to paraquat recycling in redox metabolism. Paraquat is an easily reducible organic cation, which interacts favorably with the reductive agent NADPH [127]. NADPH is a cellular electron carrier involved in many bio-reductive pathways for cellular metabolism and easily donates an electron to paraquat to become NADP+. This causes disruptions in cellular metabolism, as it depletes the NADPH pool of the cell and interrupts metabolic homeostasis. The depletion of NADPH also causes the accumulation of oxygen free radicals such as superoxide since these species are reduced by NADPH as a cytoprotective measure. The generation of free radicals causes tissue damage at the ocular surface due to the highly reactive nature of free radicals, which steal electrons from key biological molecules. On the ocular surface, a common result of free radical damage is conjunctivalization of the cornea with vascular pannus [127]. Severe injury may result in a chronically disordered ocular surface, manifesting in symptoms such as dryness, punctal stenosis, symblepharon, ankyloblpharon, forniceal shortening, entropion, and trichiasis [128,129]. Early appropriate treatment by flushing thoroughly with water may avoid highest levels of injury and minimize damage to minor corneal opacity and pannus as the main complications [130]. Paraquat-containing pesticide mixtures such as preeglox-L, which also contains diquat and surfactants, have also been linked to corneal epithelium deterioration [131].

Many herbicides contain the active ingredient glyphosate, an organophosphate compound that has toxic effects on several bodily systems. Organophosphates inhibit acetylcholinesterase (AChE), a key enzyme in the nervous system, by phosphorylating a serine hydroxyl group of its active site [132,133]. The inhibition of AChE by pesticides is known to cause eyelid muscle twitching, eye pain, and miosis [132,134]. Glyphosate has been shown to cause conjunctival irritation and superficial corneal injury, especially in cases where eye irrigation is delayed. [135,136].

Organophosphate exposure has also been linked to decreased glutathione content and increased levels of oxidative stress as measured by malondialdehyde levels in mouse eye and brain tissue upon exposure to the insecticide chlorpyrifos [137,138,139]. Cellular disruption via organophosphate pesticide exposure may result from inhibition of antioxidant enzymes such as superoxide dismutase and catalase, as well as an increase in inflammatory cytokines such as tumor necrosis factor-α (TNF-α), interleukin (IL)-6, and IL-1β [140,141,142,143,144].

Flubendamide is a newer synthetic phthalic acid diamide insecticide with low immediate toxicity to humans [145]. The effects of flubendiamide on the ocular surface were studied in non-target *Drosophila melanogaster* to evaluate cross-reactivity in species at which the insecticide is not directed. It was found that flubendiamide altered the compound eye architecture and bristle pattern orientation in four generations of non-target *D. melanogaster* at doses consistent with those administered in fields in India [146,147]. The irritative nature of flubendiamide is further explored in a report published by the Food Safety Commission of Japan, as the insecticide was linked to ocular inflammation in rats [148]. 

### 5.3. Fungicides

Mancozeb, a manganese/zinc ethylene-bis-dithiocarbamate fungicide, inhibits enzyme activity in fungi by complexing with enzymes containing sulfhydryl groups including those that participate in generation of ATP. This carbamate pesticide has been shown to cause toxic epidermal necrolysis and ocular lesions in cases of human exposure [149]. Carbamate pesticides, like organophosphate pesticides, are known to affect the AChE enzyme in human cells. Carbamates cause the carbamylation of AChE in neuronal synapses and neuromuscular junctions, and whereas organophosphates bind irreversibly to AChE, carbamates bind reversibly to the enzyme [150]. 

A study conducted at a seed supply warehouse in Japan identified n-butyl isocyanate, a hydrolyzed product of the fungicide benomyl as the cause for ocular irritation among several workers [151]. This finding has significant implications on regulatory measures for commercially used pesticides, as the safety of not only the pesticide must be taken into account but also the products of its degradation.

## 6. Workplace Ocular Injuries

### 6.1. Overview

The workplace is a common site of ocular injuries, as approximately 2000 U.S. workers experience job-related eye injuries requiring medical treatment each day [152,153]. These injuries can be divided into three broad categories: striking or scraping, penetrating, and chemical and thermal burns [154,155,156]. Striking or scraping constitutes a common type of ocular injury, and involves the ejection of small particles such as dust, wood chips, or cement chips into the ocular surface, as well as larger objects that result in blunt trauma to the eye [157]. Penetration occurs when objects such as nails, staples, or slivers of wood or metal move through the surface of the eye and potentially result in the permanent loss of vision [158,159]. Chemical and thermal burns to the eye are frequently caused by industrial chemicals and cleaning products, and welding processes respectively [154]. A cross-sectional retrospective study used de-identified data from a large-scale employer survey of individuals reported to have ocular workplace injuries in the United States between 2011 and 2018 showed the highest likelihood of this type of injury in those employed in: fishing, farming and forestry; construction; and production industries [160]. In this study, the major reasons for eye injury were contact with objects (65%) and exposure to harmful substances (26%).

### 6.2. Foreign Object Injuries

In the fishing industry and in sports fishing, injury can occur when fishing hooks, lures, rod tips, or lines accidentally strike the eye [161,162,163,164]. Any eye structure may be involved with damage ranging from corneal abrasion to penetrating injury to globe rupture. Lenses, particularly wraparound lenses can protect the eye during fishing.

**Figure 1:**
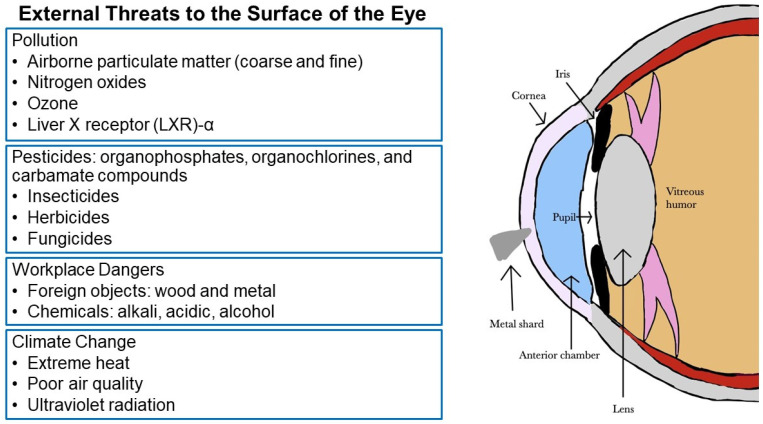
Dangers posed to the ocular surface by the environment and diagram of the anterior segment of the human eye. Key structures are labeled. A metal shard is depicted penetrating the cornea. This foreign body should be carefully removed.

Wood injuries may occur in forestry workers, wood workers, and gardeners [165]. Infections of bacterial or fungal origin are a significant risk, especially if the wood fragment is not removed promptly [166,167]. The high infection rate is attributed to the pores on the wood surface and the characteristics of organic and vegetative matter, which provide bacterial growth medium [168]. The infection may manifest as orbital cellulitis, abscess formation, and even intracranial infection. Detection of wood in the eye is challenging because it is carbon-containing and not visible on conventional x-ray may not image well on CT or MRI [169,170]. If the chip is small and on the surface, it may be flushed with eyewash; however, deeper penetration shards may require surgical intervention and antibiotic treatment (Figure 1) [171].

Metal workers are particularly susceptible to dry eye according to a study by Ai et al. [172]. They attribute the vulnerability of metal workers to dry eye disease to their exposure to dust and chemicals. In a cross-sectional study of welders in Turkey, exposure to cadmium and lead were correlated with dry eye disease [173]. Chen et al. also found lead exposure and presence of lead in tears to be associated with dry eye disease [174]. 

Metallic foreign bodies can enter the eye during use of hammer and nail, nail gun, or stapler [175,176,177,178]. Metallic foreign body removal is key in order to avoid consequences such as infection, swelling, inflammation, astigmatism, and opacification of the cornea [179]. Release of iron or copper from a retained foreign body in the eye can lead to cataracts, glaucoma, and pigment changes on the retina [180,181,182].

### 6.3. Chemical Injuries

Cleaning products used around the home and office are often formulated with chemicals that can damage the eye. Chemical burns to the eye can come from acids, alkalis, or alcohol (Table 1) [183]. Acids cause protein coagulation, which somewhat limits damage by forming a self-containing barrier while alkalis are lipophilic, cause saponification and penetrate more deeply into tissue, leading to extensive and severe damage to the cornea [184,185]. Alkali burns can result in loss of limbal epithelial stem cells that are essential for regeneration of corneal epithelium [186].

In the United States, bleaches, categorized as alkali, accounted for more than 25% of ocular exposures reported to poison control centers between January 2000 and December 2016 [187]. Bleach can cause burning sensation, tearing, photophobia, and conjunctival abrasions [188,189,190]. 

Hydrofluoric acid is a highly reactive compound used in industry and some cleaning and rust-removing products. It can cause burns, tearing, conjunctivitis, and corneal ulcers and opacification [191,192].

Exposure of the eye to ethanol, which is often used as a disinfectant, can damage corneal epithelial and stromal cells, and cause inflammation and proinflammatory cytokine release [193,194].

### 6.4. Preventing Damage from Chemicals and Foreign Bodies

Particles in the eye and chemical eye burns require immediate flushing and therefore access to water or other rinsing solutions in the workplace is essential [195]. Most occupational eye injuries are potentially preventable [196]. Eye protection needs to fully cover the eyes [197]. There are multiple forms of appropriate eye protection, some of which include goggles, face shields, and full-face respirators that reduce the likelihood of work-related eye injuries [191,198,199,200]. Indirectly vented goggles that fit from the corners of the eye across the brow provide effective protection from splashes, sprays, and respiratory droplets that may be encountered in the workplace [156]. Although goggles are viable in shielding the eyes from irritants, other parts of the face are neglected by goggles and thus remain vulnerable despite goggle usage. Face shields that wrap around the face to the ears can be utilized in addition to goggles to provide increased protection from splashes and sprays for the entire face as opposed to simply the eyes. Requiring these forms of protection in the workplace can contribute to a reduction in daily work-related ocular injuries [201,202].

## 7. Climate Change

### 7.1. Key Features of Climate Change

The Climate Change phenomenon is marked by fluctuations in temperature and precipitation patterns, flooding or drought, and increased frequency of extreme weather events, any of which can have deleterious effects on human health [203,204,205]. Some of the major climactic changes occurring include rising global temperature, increasing atmospheric CO_2_ levels, increasing sea level, glacial melting, and ozone depletion [206]. Ozone depletion has been linked to modified precipitation patterns, increased frequency of extreme precipitation events, augmented ultraviolet radiation levels at the surface of the earth, and altered temperature patterns in several regions of the globe. [207,208,209,210]. 

The Intergovernmental Panel on Climate Change 2022 Report on Impacts, Adaptation and Vulnerability outlines potential impacts on human health including food insecurity and malnutrition, anxiety and stress, increase in vector-borne disease and exposure to wildfire smoke, atmospheric dust, and allergens in the air [211].

Though the negative consequences of global warming on human well-being and their mechanisms are largely understood and highly publicized, the specific effects on the ocular surface are not nearly as well-characterized [212]. Effects of climate change on the ocular surface can occur due to photo-oxidative damage from increased ultraviolet exposure, thermal damage, and pollution effects [213]. A study conducted in Southern Spain using climate data and eye disease data has shown that climate change has increased the incidence of eye disease, representing a huge economic burden [214]. Cornea, scleral, and conjunctival pathologies were among the most affected by environmental variables in this arid region. 

The purpose of this section is to investigate the effects of climate change on ocular surface disorders, such as dry eye disease, which have cascading economic consequences on the healthcare systems of major countries worldwide.

### 7.2. Elevated Global Temperature and Increased Frequency of Extreme Heat Events

The 5 years since 2016 have ranked as the 5 warmest on record [215]. The rapid warming of the global temperature is a facet of climate change with deleterious consequences for human health [216,217]. 

Studies of the localized effect of increased global temperature on the eye have linked rising temperature to increased rates of corneal damage, cataracts, glaucoma, and retinal damage [214,218,219,220]. Increased temperatures have also been shown to increase instances of eye infections such as bacterial, fungal, and amoebic keratitis, leading to significant ocular discomfort and possible threat to vision [221,222,223]. An increase in thermal energy surrounding ocular structures has also been shown to induce an inflammatory response in the eye, as evidenced by elevated levels of inflammatory cytokines such as IL-1β and IL-6 in corneal cells [224]. The thermal damage to the structures of the eye is attributed to both an increase in the temperature of the cornea due to environmental conditions, as well as an overall increase in body temperature that results from living in a warmer climate [225]. The Dry Eye Assessment and Management (DREAM) Study looked at dry eye disease over a wide geographic area in the United States and found that corneal dryness as measured by corneal fluorescein staining was greater in semiarid and subtropical desert regions while moist climates were associated with less severe dry eye disease [226].

### 7.3. Air Quality

Elevated temperatures resulting from global warming have detrimental effects on the quality of air at the ground level [227,228]. Documented effects of climate change on air quality include smoke exposure, increased allergen content, elevated levels of air pollutants such as carbon dioxide and nitrous oxide, and augmented ground-level ozone concentrations [229]. The effects of air pollution on general eye health are well-documented, as air pollutants are known to cause symptoms ranging from minimal or no detriments to chronic discomfort and irritation [43,230]. Several Delhi-based studies investigated the effects of chronic exposure to air pollution on the ocular surface in a metropolitan context, and found an increased incidence of ocular surface disorders within individuals who traveled frequently in highly polluted regions of the city [91,231]. The positive association between ocular surface deficits and increased air pollution was supported in two California-based studies, as air pollution was found to cause significant eye irritation [232,233]. A recent study from Beijing compared ocular characteristics of subjects in heavily polluted areas to those in slightly polluted regions as measured by air quality index (AQI) and specific components encompassing particulates, NO_2_, and sulfur dioxide (SO_2_) [234]. The Ocular Symptom Disease Index (OSDI) questionnaire was used to assess eye discomfort and scores were positively correlated with AQI, PM2.5, PM10, and NO_2_ levels. Conjunctival injection and Goblet-cell density were found to correlate with AQI, PM2.5, PM10, and NO_2_. Concentration of the inflammatory cytokine IL-6 in tears was also higher in persons living in more polluted areas. 

Other air quality measures have been linked to ocular surface disorder pathogenesis and general ocular discomfort as well. For instance, ground-level ozone has been found to induce an inflammatory response on the eye surface, contributing to increased irritation in conjunctival allergic reactions and ocular discomfort [50,235]. Kim et al. performed a prospective observational study looking specifically at ground-level ozone and dry eye disease in 33 subjects and found that higher ozone exposure over a time period of only one week decreased tear secretion and increased eye discomfort [236].

### 7.4. Increased Ultraviolet (UV) Radiation

UV radiation has clear deleterious effects on human health and is a known cause of cellular damage, leading to diseases such as cancer [237]. Ocular exposure to UV radiation has different effects on individual structures of the eye, as cytoprotective measures and efficiency of repair mechanisms are specific to each region [238]. For instance, the anterior segment of the eye contains melanocytes and pigment epithelium that produce melanin which form a physical block that absorbs UV light and protects the iris [239].

The lens of the eye is very vulnerable to oxidative damage from UV exposure, but has antioxidant defense systems, both non-enzymatic, such as glutathione and ascorbic acid and enzymatic, such as superoxide dismutase, that minimize damage [240]. UV damage to the lens of the eye is of particular concern, as phototoxic reactions in the outer epithelial cells and inner fiber membrane can cause light sensitivity and alter the refractive index of the lens material [238]. These effects result from structural damage to the crystallin proteins of the eye lens, such as glycosylation of lysine residues, leading to the aggregation and crosslinking of normal lens proteins and eventual opacification of the lens into cataracts [241]. The link between UV exposure and cataract development has been established for over 40 years [242,243,244]. There is limited protein turnover within the lens of the eye; thus, the damage sustained from UV exposure in the lens accumulates over time and transparency is lost [245].

UV exposure causes keratitis of the corneal epithelial [246,247]. Acute exposure leads to photokeratitis with conjunctival hyperemia, decreased visual acuity, inflammation, and pain [248,249,250]. Fortunately, recovery is usually complete as the cornea will re-epithelialize within a few days. Chronic UV exposure can cause ocular surface disorders such as pterygium and may lead to squamous cell carcinoma of the cornea [251,252].

The conjunctiva, or the mucosal membranes that cover the eye and line the eyelids, are also susceptible to UV-induced damage. Conjunctival UV autofluorescence (UVAF) is a reliable non-invasive biomarker of preclinical damage, with high levels correlating to greater degree of outdoor sun exposure [253]. High levels of UVAF, indicating excessive UV exposure, have been associated with the pathogenesis of ocular disorders of the conjunctiva including pterygium [254,255]. Several studies have suggested implications of UV exposure in increased risk of conjunctival melanoma as well [256,257]. Exfoliation syndrome and exfoliation glaucoma, characterized by abnormal deposition of fibrillar extracellular material in the anterior chamber of the eye, although genetically based, may also be promoted by excess UV exposure [258,259]. 

Exposing the retina to UV radiation can lead to the destruction of photoreceptors, and in cases of intense exposure, the development of retinal lesions [260,261]. UV radiation can be an accelerating factor in age-related macular degeneration, the leading cause of irreversible blindness in older persons [262,263,264]. While the cornea and lens block in excess of 99% of UV radiation from passing to the retina, additional protection for the cornea and lens may be attained with UV blocking contact lenses [265].

## 8. Conclusions

The ocular surface, consisting of the cornea, limbus, conjunctiva, and tear film, is subject to unceasing contact with the environment. The integrity of the ocular surface, with maintenance of a healthy state of tears and tear film, is crucial in protecting the delicate tissues of the eye from toxic exposures. Chemicals and substances such as pesticides, cleaning products, and various pollutants that may come in contact with the eyes are tested to determine their potential to cause irritation or other ocular toxicity. Testing methods that employ cells in culture or computer analyses are designed to avoid use of animals. Toxicity to the eye surface can cause various types of tissue damage that may include edema, inflammation, and denudation of corneal or conjunctival epithelium. Although the eye surface can often self-repair when the toxin is removed, severe injury can lead to dry eye disease, corneal ulcers, cataracts, glaucoma, and even blindness. This review has discussed the ocular surface damage that can occur due to exposure to a variety of categories of chemicals and particulate matter in our environment at home, in the workplace, and in the course of everyday life (Figure 1). Recognizing, avoiding, and minimizing these exposures is central and protecting the eyes is of crucial importance. When there is possible exposure to liquid, dust, or particles, eyes should be protected with snug-fitting safety glasses or goggles. Pollution monitoring and research on the effects of air pollution on the eye surface are needed. Determining underlying mechanisms that lead to damage can improve our ability to prevent and treat exposures.

## Figures and Tables

**Table 1:** Characteristics of chemical burns to the ocular surface.

Type of Burn	Chemical Causes	Where Found
Alkali	Calcium carbonate, magnesium carbonate	Lime
Alkali	Calcium hydroxide	Plaster, mortar, cement
Alkali	Sodium hydroxide	Drain cleaner
Alkali	Potassium hydroxide	Caustic potash, liquid fertilizer, soft soaps
Alkali	Magnesium hydroxide	Fireworks, sparklers
Alkali	Ammonium hydroxide	Cleaning agents, fertilizers, window cleaner
Alkali	Sodium tripolyphosphate	Dish detergent, kitchen and bathroom cleaners
Acidic	Hydrofluoric acid	Glass polisher, rust remover, industrial cleaners
Acidic	Hydrochloric acid	Food and leather-processing compounds, swimming pools
Acidic	Sulfuric acid	Toilet cleaner, battery fluid
Acidic	Sodium hypochlorite, calcium hypochlorite	Bleach, pool cleaners
Acidic	Acetic acid	Vinegar
Alcohol	Ethanol	Hand sanitizer
Alcohol	Methanol	Industrial solvents, pesticides
Alcohol	Isopropanol	Antifreeze, disinfectants, antiseptics

## Data Availability

Not applicable.
